# ELIMINATOR: essentiality analysis using multisystem networks and integer programming

**DOI:** 10.1186/s12859-022-04855-z

**Published:** 2022-08-06

**Authors:** Asier Antoranz, María Ortiz, Jon Pey

**Affiliations:** 1 Intelligent Biodata Ltd, San Sebastian, Spain; 2 BMS Center for Innovation and Translational Research Europe (CITRE), A Bristol Myers Squibb Company, Seville, Spain

**Keywords:** Gene essentiality analysis, Constrain-based modelling, Multisystem networks, In-silico methods

## Abstract

A gene is considered as essential when it is indispensable for cells to grow and replicate in a certain environment. However, gene essentiality is not a structural property but rather a contextual one, which depends on the specific biological conditions affecting the cell. This circumstantial essentiality of genes is what brings the attention of scientist since we can identify genes essential for cancer cells but not essential for healthy cells. This same contextuality makes their identification extremely challenging. Huge experimental efforts such as Project Achilles where the essentiality of thousands of genes is measured together with a plethora of molecular data (transcriptomics, copy number, mutations, etc.) in over one thousand cell lines can shed light on the causality behind the essentiality of a gene in a given environment. Here, we present an in-silico method for the identification of patient-specific essential genes using constraint-based modelling (CBM). Our method expands the ideas behind traditional CBM to accommodate multisystem networks. In essence, it first calculates the minimum number of lowly expressed genes required to be activated by the cell to sustain life as defined by a set of requirements; and second, it performs an exhaustive in-silico gene knockout to find those that lead to the need of activating additional lowly expressed genes. We validated the proposed methodology using a set of 452 cancer cell lines derived from the Cancer Cell Line Encyclopedia where an exhaustive experimental large-scale gene knockout study using CRISPR (Achilles Project) evaluates the impact of each removal. We also show that the integration of different essentiality predictions per gene, what we called Essentiality Congruity Score, reduces the number of false positives. Finally, we explored our method in a breast cancer patient dataset, and our results showed high concordance with previous publications. These findings suggest that identifying genes whose activity is fundamental to sustain cellular life in a patient-specific manner is feasible using in-silico methods. The patient-level gene essentiality predictions can pave the way for precision medicine by identifying potential drug targets whose deletion can induce death in tumour cells.

**Supplementary Information:**

The online version contains supplementary material available at 10.1186/s12859-022-04855-z.

## Introduction

We can define an essential gene as a gene whose activity is fundamental to sustain life [1]. It is precisely the critical importance of these genes that brings the attention of scientists. For instance, in cancer research, specific essential genes of this condition are considered as promising drug targets as their deletion can induce death in tumour cells [2].

The essentiality of a gene is not a structural property, it depends on the biological scenario under consideration [3], including the cellular environmental conditions, disease phenotypes, etc. The contextual dependency of essential genes makes their experimental identification an extremely difficult task. The huge effort of experimental initiatives such as Project Achilles in creating an archive of essential genes is of utmost interest to the scientific community [2]. However, the biological context in which a particular gene turns out to be essential is exceptionally critical in cancer, where the essentiality of a gene could emerge at patient level [4]. This highlights the role of in-silico gene essentiality identification approaches that effectively integrate -omics datasets to contextualize a given biological scenario.

During the last decade, many successful examples have been presented on integrating omics datasets with biological networks in the context of efficient mathematical models to address an assortment of biomedical problems [5], including the identification of essential genes [6]. We can find relevant insights provided by these algorithms in different fields, ranging from microbiology [7] to cancer research [8], among others.

Despite the recent advent of machine-learning based gene essentiality analyses [9, 10], traditionally, approaches referred to as Constraint-Based Modelling (CBM) led the field setting the foundations for the development of different methodologies to predict essential genes [11–13]. In essence, CBM integrates omics data in the context of genome-scale metabolic networks resulting in a linear system of inequalities. The arising system of inequations is usually solved using linear optimization techniques [14]. Here, essential genes emerge from their indispensability when ensuring the activity of an artificial metabolic reaction, referred to as biomass reaction, which involves the metabolic requirements of the cell for its replication [15].

CBM has been also applied on signalling networks and gene regulatory networks with either gene-expression or proteomic data [16, 17]. In this work, we extend the ideas in traditional CBM by going beyond signalling and metabolism considering multisystem networks [18]. In addition, and in analogy with CBM, here we identify genes whose activity is essential for a relevant biological task. Thus, the emerging set of essential genes will be richer and more diverse than in traditional CBM, capturing a variety of biological processes [19].

Overall, this article introduces a new methodology for the in-silico identification of essential genes. This approach combines three main inputs: (i) An indispensable biological entity/process required to sustain cellular life, (ii) a set of interaction networks including the molecular requirements to activate the aforementioned indispensable entity and (iii) an experimental dataset that reflects, at least qualitatively, the genetic landscape of the sample/patient, e.g., gene expression data.

These inputs are subsequently encoded into a mathematical model (Integer Linear Program, ILP) [20] that finds the minimum number of lowly expressed genes required to activate the given relevant function. Then, a systematic approach identifies artificial gene knockouts that lead to require additional unexpressed genes to activate the critical biological entity/process. These knockouts are precisely considered as *essential genes*. This is further illustrated in the manuscript through a series of toy examples. In addition, we successfully validated a continuous score representing the degree of essentiality of a given gene, referred to as the *Essentiality Congruity Score*. We also show the relevance of each of these inputs by evaluating the performance of the method in a different set of scenarios. Finally, we apply the methodology to a group of breast cancer patients and subsequently support the relevance of the emerging essential genes based on a literature review.

## Methods

In the following section, we introduce the in-silico gene-essentiality framework presented in this article. In the first subsection, referred to as *Pathways*, we describe the biological pathway compendium used for the model; in the second subsection, mentioned as *Cell lines and samples*, we describe all the experimental data used throughout the study; the third subsection, *Mathematical model*, describes the mathematical equations modelling the pathways and integrating the experimental data; and, in the fourth subsection, called *gene essentiality analysis*, we present the pipeline that systematically find essential genes. Moreover, we present the *Essentiality Congruity Score*, which assigns a quantitative value to an otherwise binary score to represent the essentiality of a gene.

### Pathways

As in Vaske et al. [19], we consider a set of well-curated pathways from the (NCI-PID) [18] database, which are represented in the UCSC Pathway Tab Format. Vaske and co-workers provided further details about the characteristics of these pathways, including their consistency when capturing cancer related knowledge. In essence, these pathways comprise vertices and edges representing various types of biological entities and their interactions respectively. For instance, vertices could denote a gene/protein, protein complex or biological abstracts like “mitosis” or “cell motility”, among others, whilst edges represent activations/inhibitions or member/component associations [19].

Following the UCSC Pathway Tab Format [19], we will consider the following interactions: member, component, activation and inhibition. As will be introduced in the next subsection, each one of these interactions is modelled by a specific set of mathematical equations.

In total, the collection of NCI-PID pathways included 2,210 proteins distributed in 175 pathways. From these pathways, 49 were labelled as tumorigenic, 6 as anti-tumoral, and 26 as unclear or tissue specific. 94 pathways were not annotated due to lack of variance on the results (no predictions of essentiality). Proteins were represented by their protein encoding genes in HGNC format.

### Cell lines and samples

Throughout the study, we used three different datasets that are further described below. Table 1 summarises the main characteristics of each listed dataset.

**Table 1:** Dataset description

Dataset	Data type	# samples	# genes
CCLE	Gene expression	917 cell lines (478 in common with Achilles	23,521 (HGNC format)
Achilles	Essentiality scores	739 cell lines (478 in common with CCLE)	18,333 (HGNC format)
GSE65194	Gene expression	178 (153 samples, 11 normal, 14 TNBC cell lines)	23,520 (HGNC format)

Dataset identifier (Dataset), type of data (Data type), number of samples (# samples), and genes (# genes) analyzed

Gene expression data from the Cancer Cell Line Encyclopedia (CCLE) [21]. The CCLE represents a compilation of gene expression, chromosomal copy number and massively parallel sequencing data from nearly 1,000 human cancer cell lines. The gene expression data for the cell lines was obtained from the Gene Expression Omnibus (GSE36133) which includes 917 cell-lines annotated with 23,521 gene identifiers (HGNC format). Gene expression data was binarized (1 expressed, 0 not expressed) using The Gene Expression Barcode 3.0 [22, 23]. Probes were mapped to HGNC identifiers (GPL570, Affymetrix Human Genome U133 Plus 2.0 Array).

Gene essentiality scores from the Achilles project [24]. Project Achilles is a systematic effort aimed at identifying and cataloguing gene essentiality across hundreds of genomically characterized cancer cell lines. These gene essentiality scores are obtained from CRISPR knockouts (CERES method) [25] on several of the cell lines included in the CCLE [21]. Achilles scores represent gene essentiality, the more negative the score, the more essential the knockout of the gene is for a given cell-line. For this analysis, we defined a gene as essential for a given cell-line if its Achilles score was below -0.5 [26]. The Achilles Essentiality Scores were downloaded from the DepMap portal (https://depmap.org/portal/download/, version 20Q1) which contained essentiality scores for 18,333 genes in 739 cell lines, 478 of which were in common with the CCLE.

Gene expression data from Breast Cancer patient samples [27, 28]. This dataset includes 178 array samples: 153 breast cancer samples (55 TNBC; 39 Her2; 30 Luminal B and 29 Luminal A), 11 normal breast tissue samples and 14 TNBC cell lines. Data production involved different array batches and hybridation series which were accounted for in the pre-processing of the data. Processed gene expression data and sample meta-data was obtained from the Gene Expression Omnibus (GSE65194). Samples belonging to cell lines were removed from further analysis. Gene expression data was discretized using The Gene Expression Barcode 3.0 [22, 23]. Probes were mapped to 23,520 HGNC identifiers (GPL570, Affymetrix Human Genome U133 Plus 2.0 Array).

### Mathematical model

**Fig. 1:**
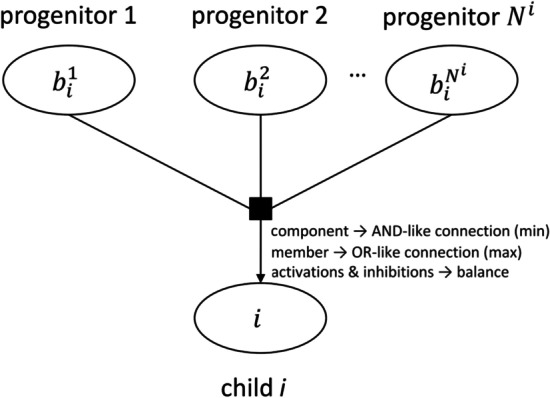
Conversion of UCSC Pathway Tab Format to valid pathways for the mathematical model. These pathways model the relationship between the *i*-th child and its progenitors using various types of interactions including component>, member>, and activations & inhibitions

The network format described above can be translated into a series of Boolean rules. However, the inherent complexity of these rules grows exponentially when regular-sized pathways are considered. In this subsection, we present the Integer Linear Programming framework (ILP) able to contend with complex networks and capturing all the essence of the Boolean rules. The ILP constitutes the core of the methodology. The mathematical equations in the ILP arise precisely from the structure of the pathway and its different interactions (Fig. 1). In essence, it calculates, first the minimum number of lowly expressed genes required to activate a given biological function necessary to sustain cellular life (referred to as wild-type solution). Then, it produces in-silico knockouts for each highly expressed gene and recalculates this number. If the number of lowly expressed genes needed to be active is larger after the in-silico knockout, the knocked-out gene is predicted to be essential for that biological function. The reader should note that genes predicted to be essential belong exclusively to the highly expressed category, a criterion well-established in the literature [29].

Let ***B***^***i***^ represent the set of all the parents for a given entity *i*. Let *N*^*i*^ be the cardinality of ***B***^***i***^, namely *N*^*i*^ =*|****B***^***i***^*|*. We can define *E*_*i*_ as a binary variable {0,1} that represents the activation status of *i* (*E*_*i*_ = *0* if inactive, *E*_*i*_ = *1* if active) in a given set of experimental conditions (wild-type or knockout). The reader should note that *E*_*i*_ is not the same as the experimental expression of the genes, the experimental data will be included later in the model. In particular, the method focuses on minimizing the number of lowly expressed genes being active but does not apply direct restrictions to expressed genes.

Now we will proceed to mathematically define the constraints based on the nature of the interaction between the *i*-th child and its progenitors.

#### Component

In analogy to the AND-like connection considered for components of a complex in Vaske et al*.,* [19], the final activation status of the child (*E*_*i*_) is determined by the minimum value from all its components. So, *E*_*i*_ = *1* if, and only if, *E*_*b*_ = *1 ∀ b ∈ ****B***_***i***_. Otherwise, we impose that *E*_*i*_ = *0*.

1 $$E_{i} \ge \mathop \sum \limits_{{\forall b \in {\varvec{B}}^{{\varvec{i}}} }} E_{b} - \left( {N^{i} - 1} \right)$$

2 $$N^{i} \cdot E_{i} \le \mathop \sum \limits_{{\forall b \in {\varvec{B}}^{{\varvec{i}}} }} E_{b}$$

where N^i^-1 represents the number of parents of the child *i.*

#### Member

The final status of node *I* is determined by the maximum value from all its members. So *E*_*i*_ = *0* if, and only if, *E*_*b*_ = *0 ∀ b ∈ ****B***_***i***_. Otherwise, we impose that *E*_*i*_ = *1*. Note the similarity with [19] where members are modelled in a OR-like fashion.

3 $$N^{i} \cdot E_{i} \ge \sum\limits_{{\forall b \in {\mathbf{B}}^{i} }} {E_{b} }$$

4 $$E_{i} \le \mathop \sum \limits_{{\forall b \in {\varvec{B}}^{{\varvec{i}}} }} E_{b}$$

#### Activations & inhibitions

The final status of the target is determined by a balance between all its activators and inhibitors. For simplicity, we can define an intermediate variable *F*_*i*_* ∈ ℤ* that expresses the activation/inhibition state of *i*,

5 $$F_{i} = \mathop \sum \limits_{{\forall b \in {\varvec{J}}_{{\varvec{i}}} }} E_{b} - \mathop \sum \limits_{{\forall b \in {\varvec{I}}_{{\varvec{i}}} }} E_{b}$$

where ${\varvec{J}}_{{\varvec{i}}}$ and ${\varvec{I}}_{{\varvec{i}}}$ represent the set of activators and inhibitors of *i*, respectively. The activation status of the child *i* is then determined by its activation state,

6 $$M\cdot\left( {E_{i} - 1} \right) \le F_{i} - w$$

where *M* is an auxiliary positive large integer (*M* = *1,000*) and *w* the relative weight between activators and inhibitors that modules the sign of $F_{i}$. Here, we considered an arbitrary value of *w* > *0,5*. The reader should note how Eq. 6 forces *E*_*i*_ = *0* when *F*_*i*_ < *w* and does not constrain *E*_*i*_ when *F*_*i*_ ≥ *w*. That is, an inhibitory state of *i* is sufficient for the inhibition of the node, while an activation state of *i* is necessary for its activation. *F*_*i*_ is precisely defined by the activation/inhibition configuration of the progenitors of *i* (see Eq. 5).

The role played by the auxiliary variable M allows that when *F*_*i*_ < *w*, the only possible solution leading to a more negative value in the left-hand-side (LHS) of Eq. 6 is, precisely, with *E*_*i*_ = *0* (remember the binary nature of *E*_*i*_). In addition, given that M is a very large integer, it will automatically lead to a very negative value, particularly -M, in the LHS of this equation, allowing any feasible difference between *F*_*i*_ and *w*. Similarly, when *F*_*i*_ ≥ *w*, Eq. 6 will be always satisfied, no matter what the value of *E*_*i*_ is. Note that Eq. 6 is only imposed when the target *i* is an abstract or a complex because the genes and proteins generally represent the entries of the network, and their global activators-inhibitors scenario are often not properly captured in individual pathways.

#### Artificially activating an abstract/complex

We will impose the activation of relevant biological functions. To that end, we define the set of all entities required to sustain cellular life as ***A***, from now on defined as actives, and an independent problem is defined for each of them.

7 $$E_{a} = 1,\forall a \in {\varvec{A}}$$

where $E_{a}$ represents the activity of the entity *a*. In practice, for a given pathway, the set **A** consists of all its abstracts and complexes.

#### Minimizing the number of lowly expressed

Let ***L*** represent the set of lowly expressed genes. $\forall a \in {\varvec{A}}$, we define the optimal solution as the one that directly minimizes the number of lowly expressed genes active in the final solution whilst $E_{a} = 1$. Note that the model will provide a specific value of the objective function for each *a ∈ ****A***. We will refer to this solution as $S_{a}^{wild}$*.*

8 $$S_{a}^{wild} = min\mathop \sum \limits_{{\forall i \in {\varvec{L}}}} E_{i} ,\forall a \in {\varvec{A}}$$

The reader should note the difference between the in-silico meaning of active genes (genes that belong to the solution) and the experimental meaning of highly expressed genes. These two definitions are not equivalent and active genes can belong to both the highly expressed and lowly expressed category.

### Gene essentiality analysis

Let ***G*** represent the set of expressed genes. $\forall a \in {\varvec{A}}$, we model each gene removal ($g \in {\varvec{G}}$) sequentially to quantify the biological impact of its knockout for a given abstract and experimental picture. The gene removal is basically imposed by forcing *E*_*g*_ to be equal to zero (*E*_*g*_ = *0*) with $g$ representing the gene that is being knocked out. Note that genes that appear in the pathway models and are not experimentally measured are considered as expressed and therefore we include them in the knock-out process.

**Fig. 2:**
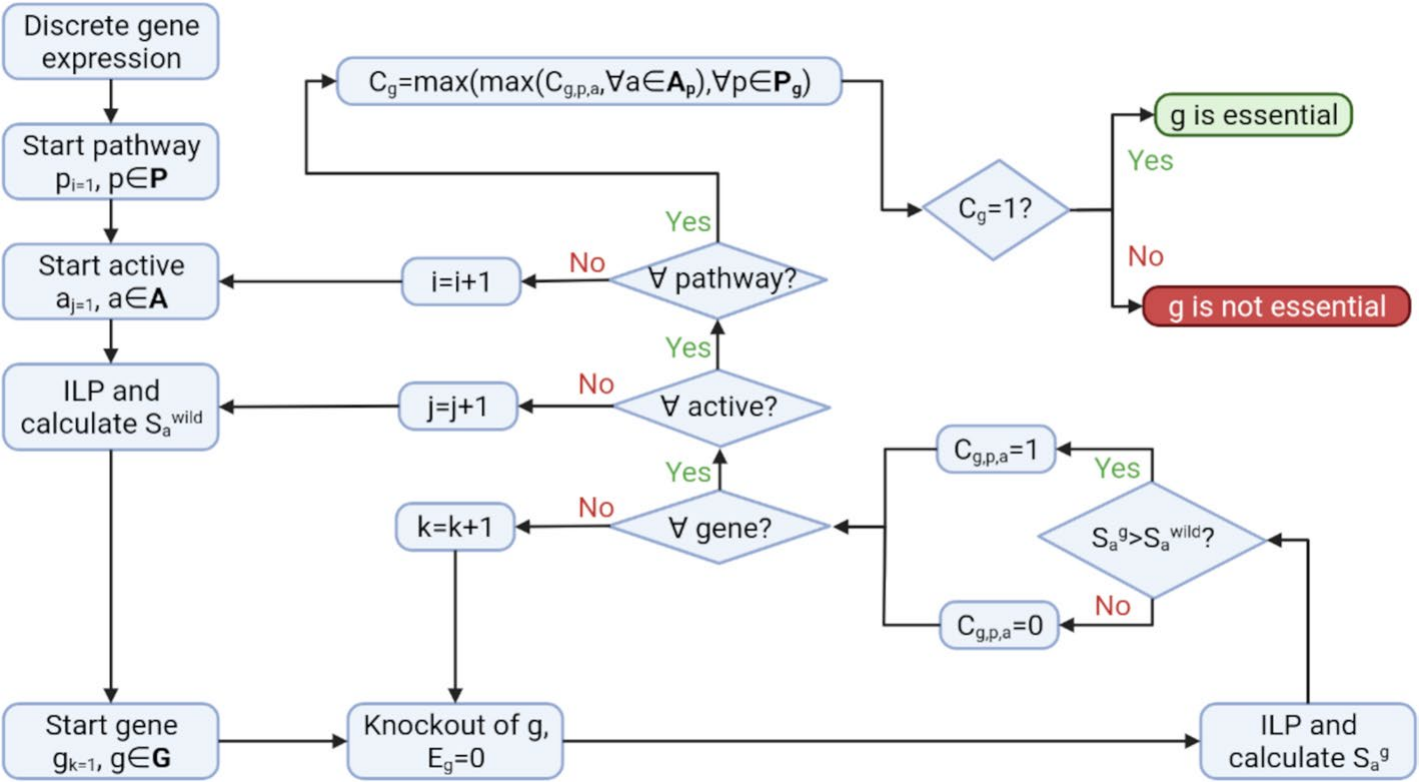
Flow diagram of the methodology. Starting from a specific experimental picture (discrete gene expression), we calculate the minimum number of lowly expressed genes required to be active for the cell to sustain cellular life ($S_{a}^{wild}$). Then, we systematically knock-out one by one all the expressed genes *g* present in the pathway **P** (Eg = 0) and recalculate the minimum number of lowly expressed genes required to be active for the cell to sustain cellular life ($S_{a}^{g}$). We define a gene as essential for a given active if $S_{a}^{g} > S_{a}^{wild}$. We repeat this process for all the genes, actives, and pathways included in the database. The essentiality of a gene g is finally defined as the maximum of all its essentiality predictions across all actives **A** and pathways where the gene appears **P**_**g**_

Afterwards the problem is solved (Eq. 8) and the minimum number of lowly expressed genes active is calculated ($S_{a}^{g}$). If $S_{a}^{g} > S_{a}^{wild}$ the gene is considered as essential for the cell to carry out that biological process (*a*) in the given pathway. Else, the gene is considered as not essential. In other words, if the new solution $S_{a}^{g}$ modelling the knockout of gene g requires the presence of more lowly expressed genes that the wild type $S_{a}^{wild}$, we assume that the removal of g is causing a significant impact to the phenotype represented by the gene expression dataset. The flow diagram corresponding to the methodology is summarised in Fig. 2.

Note that the proposed methodology, analyses every pathway independently and solves the optimization problem for every gene and every active (gene complex or biological abstract) in the pathway, as illustrated in Fig. 2. This means, that for a given gene, multiple predictions of essentiality can be produced in the same pathway (as many as there are elements in ***A*** for that pathway). Conceptually, our method assumes that if the gene is essential for at least one entity required to sustain cellular life (active), then its knockout would be fatal for the cell overall. Therefore, a gene is essential for a pathway, if it is essential for any of its actives.

9 $$C_{g, p} = {\text{max}}\left( {C_{g,p,a} ,\forall a \in {\varvec{A}}} \right)$$

where $C_{g,p,a}$ is a binary variable ($C_{g,p,a} \in \left\{ {0,1} \right\}$) that represents the essentiality of the gene *g* in the pathway *p* for the entity *a*.

Moreover, different pathways are not completely disjoint sets and often have common genes. This means, that we can have more than one prediction of essentiality for a gene in different pathways. Similarly, we assume that if the gene is essential for at least one pathway, then its knockout would be fatal for the cell overall. Therefore, a gene is essential, if it is essential for any of its pathways.

10 $$C_{g} = {\text{max}}\left( {C_{g,p} ,\forall p \in {\varvec{P}}_{{\varvec{g}}} } \right)$$

where ${\varvec{P}}_{{\varvec{g}}}$ represents the set of pathways where the gene *g* is present.

#### Globally essential and globally not essential genes

If the knockout of a gene *g* leads to $C_{g} = 1$ for each of the experimental datasets, the gene is considered globally essential. Similarly, if the knockout of a gene leads to $C_{g} = 0$ for every experimental dataset, the gene is considered globally not essential. Both globally essential and globally not essential genes are excluded from downstream analysis. Given the ulterior motives of the method, we are particularly interested in genes whose essentiality depends on the experimental dataset. Therefore, if a particular gene turns out to be essential in a cancer phenotype but not in the corresponding healthy tissue, we can identify it as a potential drug target.

#### Essential Congruity Score (ECS)

The proposed methodology assumes that predictions of essentiality ($C_{g,p,a} = 1$) are more impactful than predictions of no essentiality ($C_{g,p,a} = 0$) and the essentiality of a gene *g* is defined as the maximum of all its predictions (Eqs. 9 and 10). This assumption, however, is very susceptible to false positive predictions (not essential genes predicted as essential) that can have a huge influence in the obtained results. To address this issue, we defined the *Essential Congruity Score* ($ECS$) as:

11 $$ECS_{g} = \frac{{\mathop \sum \nolimits_{{\forall p \in {\varvec{P}}_{{\varvec{g}}} }} \left( {\mathop \sum \nolimits_{{\forall a \in \overline{{{\varvec{A}}_{{\varvec{p}}} }} }} C_{g,p,a} } \right)}}{{\mathop \sum \nolimits_{{\forall p \in {\varvec{P}}_{{\varvec{g}}} }} \left( {\left| {{\varvec{A}}_{{\varvec{p}}} } \right|} \right)}}$$

where $ECS_{g}$ is the Essential Congruity Score for the gene *g*, ${\varvec{P}}_{{\varvec{g}}}$ represents the set of pathways in which the gene *g* is present, $\overline{{{\varvec{A}}_{{\varvec{p}}} }}$ is the set of actives for the pathway *p* with at least one prediction of essentiality, and $C_{g.p,a}$ is the prediction of essentiality for the gene *g*, in the pathway *p*, and for the active *a*. $ECS_{g} = 0$ means that in none of the instances the gene *g* was predicted as essential while $ECS_{g} = 1$ means that in 100% of the predictions the gene *g* was essential.

## Results

In this section, we show the results obtained with the proposed methodology in a different set of scenarios: (1) a simple toy example showing the key conceptual aspects of the methodology and the functioning of the equations; (2) a case study using the gene essentiality data from the Achilles project illustrating the biological validity of the obtained results; (3) a breast cancer dataset which results are validated in the literature.

### Toy example

**Fig. 3:**
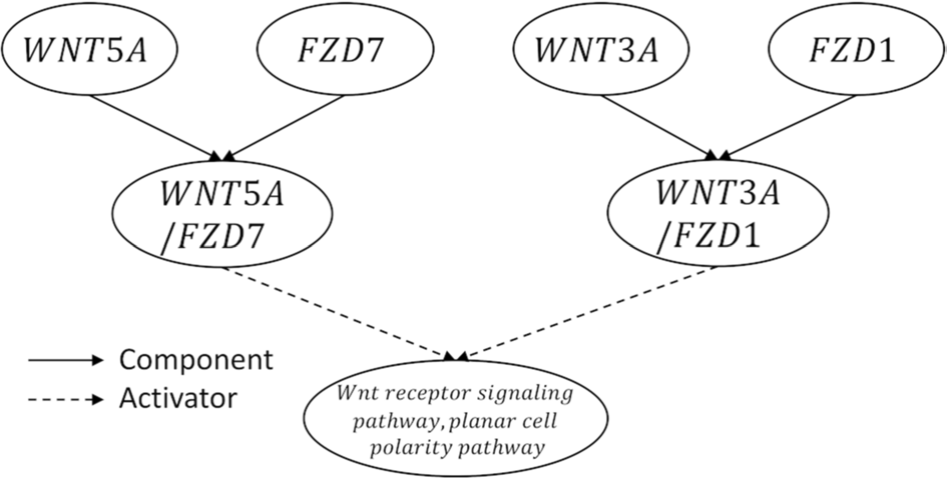
Toy example. Graphical representation of the pathway activating the Wnt receptor signaling pathway, planar cell polarity pathway. Component-type interactions are represented with solid arrows whilst activation-type interactions are illustrated with dashed lines

First, we considered a simplification of the *Wnt receptor signaling pathway, planar cell polarity pathway*, which is shown in Fig. 3 [18]. The simplified subnetwork comprises four genes (WNT5A, FZD7, WNT3A and FCD1), two complexes (WNT5A/FZD7 and WNT3A/FZD1) and one abstract (Wnt receptor signaling pathway, planar cell polarity pathway). As mentioned earlier, the methodology comprises two main steps: (i) calculating the minimum number of lowly expressed genes that we need to activate in order to trigger a given active *a* ($S_{a}^{wild}$) and (ii) performing an exhaustive in-silico gene knockout to find gene deletions that unavoidably lead to the need of activating extra lowly expressed genes in order to trigger the given entity ($S_{a}^{g} > S_{a}^{wild}$).

In the forthcoming lines we will define three scenarios based on simulated data. These scenarios show different solutions based on whether WNT5A and WNT3A are expressed or not while FZD7 and FZD1 are always expressed i.e., FZD7 & FZD1 ∈ **G**. Table 2 summarises the solution of the different proposed scenarios. The complete solution of the mathematical model for each scenario is included in Additional file 1.

**Table 2:** Toy example solution

Scenario	Gene expression				Entity activity						
	WNT5A	FZD7	WNT3A	FZD1	WNT5A	FZD7	WNT3A	FZD1	W5F7C	W3F1C	ABSTR
A	0	1	0	1	0	0	1	1	0	1	1
B	0	1	1	1	0	0	1	1	0	1	1
C	1	1	0	1	1	1	0	0	1	0	1
A′	0	1	0	1	1	1	0	0	1	0	1
B′	0	1	1	1	0	0	1	1	0	1	1
C′	1	1	0	1	0	0	1	1	0	1	1

Possible scenarios when FZD7 and FZD1 are expressed. For each scenario, the expression values of each gene and the activity values of each entity are included. W5F7C represents the WNT5A/FZD7 complex, W3F1C represents the WNT3A/FZD1 complex, and ABSTR represents the Wnt receptor signaling pathway, planar cell polarity pathway. (A) WNT5A and T3A are not expressed. For the abstract to be active we need to activate one lowly expressed gene (WNT3A in the example). (B) WNT5A is not expressed and WNT3A is expressed. For the abstract to be active we do not need to activate any lowly expressed gene. (C) WNT5A is expressed and WNT3A is not expressed. For the abstract to be active we do not need to activate any lowly expressed gene. (A′) Scenario A after FZD1 is knocked-out. For the abstract to be active we need to activate one lowly expressed gene (WNT5A in the example). (B′) Scenario B after FZD7 is knocked-out. For the abstract to be active we do not need to activate any lowly expressed gene. (C′) Scenario C after FZD7 is knocked-out. For the abstract to be active we need to activate one lowly expressed gene (WNT3A)

#### Minimum number of lowly expressed genes required to activate an entity

**Fig. 4:**
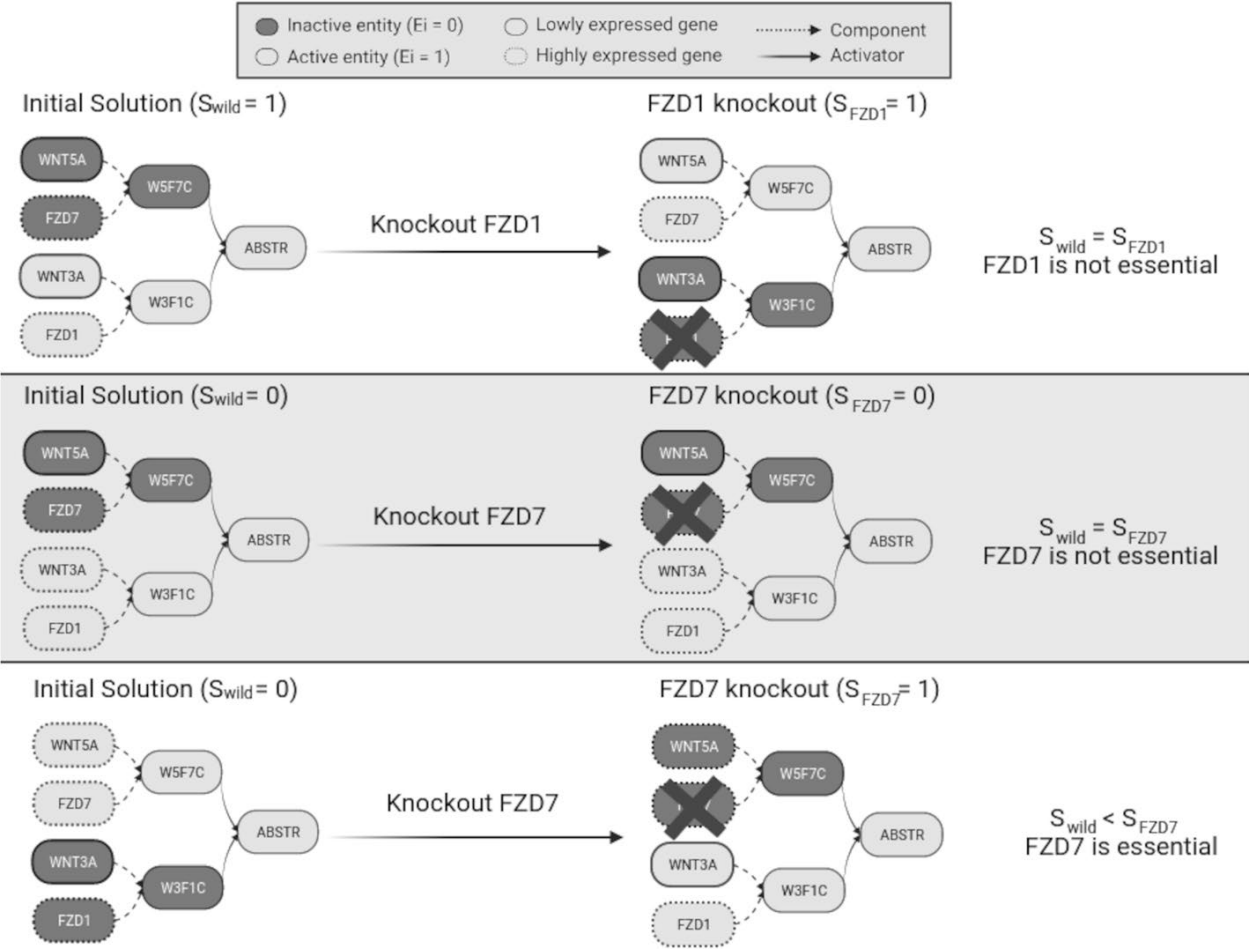
Toy example solution. Possible scenarios when FZD7 and FZD1 are expressed. W5F7C represents the WNT5A/FZD7 complex, W3F1C represents the WNT3A/FZD1 complex, and ABSTR represents the Wnt receptor signaling pathway, planar cell polarity pathway. Dark and light nodes represent inactive and active nodes in the final solution respectively, namely Ei = 0 and Ei = 1. The dashed edge in a gene g represents highly expressed genes (g ∈ **G**) whereas continuous edges represent lowly expressed genes (g ∈ **L**). **A** WNT5A and WNT3A are lowly expressed. For the abstract to be active we need to activate one lowly expressed gene (WNT3A in the example). A knockout of FZD1 requires the activation of one lowly expressed gene (WNT5A in the example) thus providing an equivalent solution (S_wild_ = S_FZD1_, FZD1 is not essential). **B** WNT5A is lowly expressed and WNT3A is highly expressed. For the abstract to be active we do not need to activate any lowly expressed gene. A knock-out of FZD7 does not require the activation of any lowly expressed gene for the abstract to be active (S_wild_ = S_FZD7_, FZD7 is not essential). **C** WNT5A is highly expressed and WNT3A is lowly expressed. For the abstract to be active we do not need to activate any lowly expressed gene. A knock-out of FZD7 requires the activation of one lowly expressed gene (WNT3A) for the abstract to be active (S_wild_ < S_FZD7_, FZD7 is essential)

The scenario shown in Fig. 4A has two expressed genes (FZD7 and FZD1) and two lowly expressed genes (WNT5A and WNT3A). For the abstract to be active, one of the two complexes needs to be active. The condition for either complex is that both of its gene components need to be active. Thus, in scenario A we need to activate one lowly expressed gene (WNT5A or WNT3A) for the abstract to be active ($S_{Abstract}^{wild} = 1$). Scenarios B and C do not require the activation of any lowly expressed gene to activate the abstract and therefore $S_{Abstract}^{wild} = 0$ (for the complete solution, please refer to Additional file 1).

#### In-silico* exhaustive gene knockout*

In the scenario shown in Fig. 4B, a knock-out in FZD7 $(E_{FZD7} = 0)$ does not require the activation of any lowly expressed gene because the abstract can be activated through the WNT3A/FZD1 complex and both its components are expressed, that is $S_{Abstract}^{wild} = S_{Abstract}^{g} = 0$. Therefore, in this scenario, FZD7 is not an essential gene. On the other hand, if we consider the scenario described in Fig. 4C, a knockout in FZD7 means that the WNT5A/FZD7 complex cannot be active, and thus the abstract needs to be activated via the WNT3A/FZD1 complex which requires the activation of one lowly expressed gene (WNT3A). In this scenario, $S_{Abstract}^{g} = 1$, while $S_{Abstract}^{wild} = 0$ and therefore FZD7 is considered an essential gene.

### Method validation

**Fig. 5:**
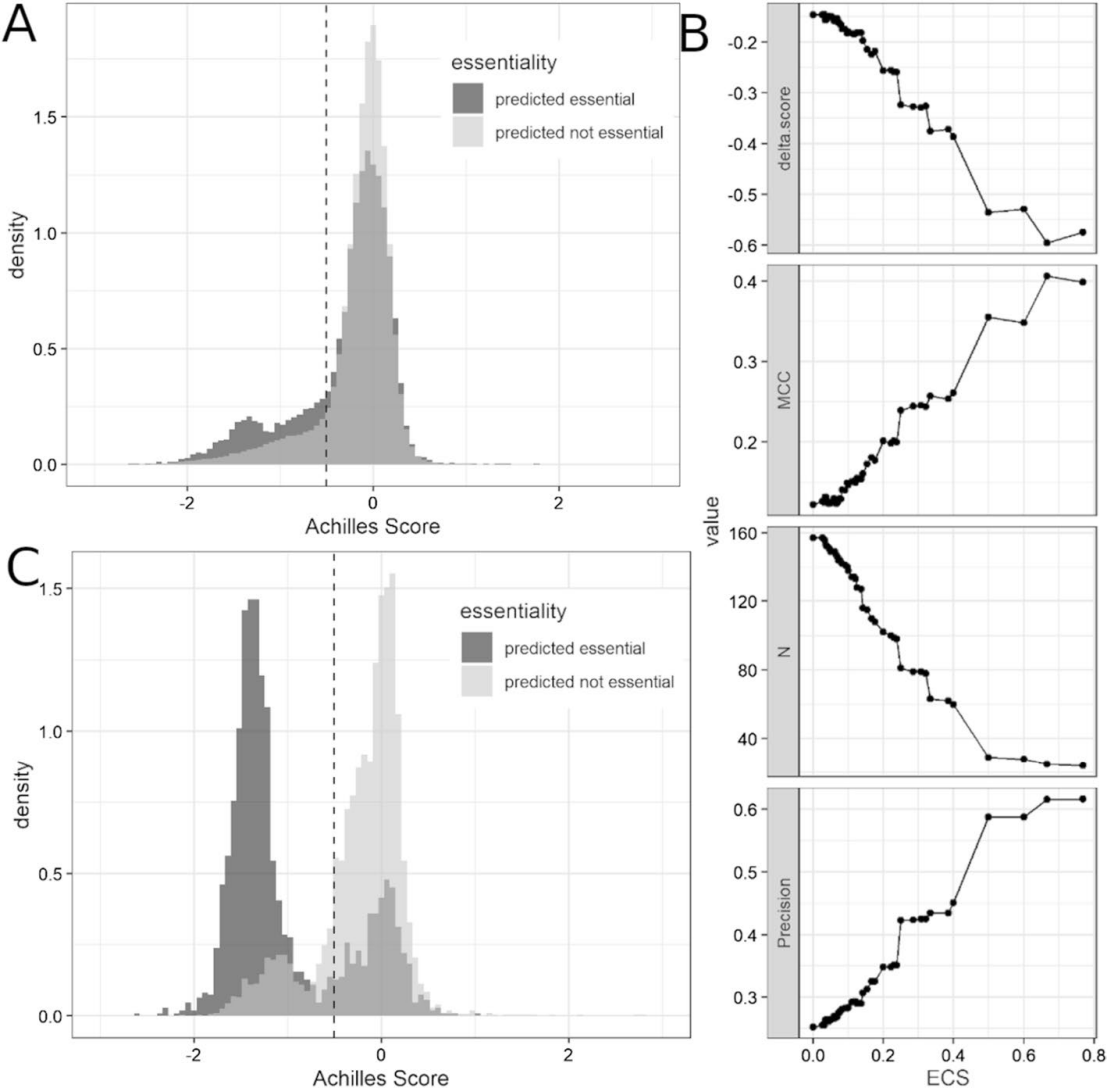
Method validation. **A** Histogram showing the results from the validation of the method. The dark distribution shows the Achilles scores of those pair gene & cell-lines predicted as essential; the light distribution shows the Achilles scores of those predicted as not essential. Genes predicted as essential have significantly lower Achilles score than genes predicted as not essential (p-value = 6.4032 × 10^–246^). The average difference between both distributions is defined by the parameter delta.score = − 0.1463. **B** Impact of ECS in the performance of the method**.** Evolution of the results when different thresholds of ECS are used to define a gene as essential. delta.score: average difference in Achilles score between the genes predicted as essential and the genes predicted as not essential; MCC: Matthew’s Correlation Coefficient; N: number of genes included in the comparison; Precision: obtained precision assuming as real essential genes those with an Achilles score < − 0.5. **C** Histogram when MCC finds its maximum (ECS = 0.6667). The average difference in Achilles Score between genes predicted as essential and genes predicted as not essential becomes bigger (delta.score = − 0.5954) and so does their significance (p-value = 0)

To validate the biological relevance of the gene essentiality predictions of our method, for a given cell line, we compared the Achilles scores of the genes *g* predicted as essential (*Cg* = 1) versus the scores of the genes predicted as not essential (*Cg* = 0) (Fig. 5A). For this analysis, globally essential (genes predicted as essential in all cell lines) and globally not essential genes (genes not essential in all the cell lines) were not included in the analysis (Methods section). This reduced the number of genes included in the comparison to 159.

Figure 5A shows how the genes predicted as essential have a significantly lower Achilles score than the genes predicted as not essential (p-value = 6.4032^–246^). The results illustrated in Fig. 5A follow the definition of essentiality represented in Eqs. 9 and 10 of the methods where a gene is considered essential if is predicted as essential for any active in any of the pathways where it appears. However, the ECS defined in Eq. 11 is a continuous score (*ECS* ∈ [0,1]) and allows to describe flexible threshold when defining the essentiality of a gene. For example, we can define genes as essential if their ECS is larger than a given threshold *th* (*Cg* = 1, *if ECSg* > *t*ℎ). We studied the impact of applying different thresholds to the ECS by evaluating the evolution of the obtained results (Fig. 5B). For this analysis, we defined a gene as essential for a given cell-line if its Achilles score was below − 0.5 [26].

Figure 5B shows how as the minimum ECS required to consider a gene as essential increases, so does the quality of the predictions. Most of the statistics shown in the different subfigures improve their performance when more demanding values of ECS are needed to define a gene as essential. We defined as the optimal cut-off the ECS where the MCC parameter finds its maximum (ECS = 0.67, MCC = 0.41, Fig. 5C). We selected the MCC because it has been proven to be the most robust metric for imbalanced data [30]. However, as the minimum required threshold increases, so does the number of genes considered globally not essential which decreases the number of genes included in the analysis (represented by N). Figure 5B also shows the monotonically increasing behaviour of the Precision curve when the minimum required ECS to define essentiality increases. This is particularly interesting for reducing experimental validation costs, as we want to make sure that genes predicted as essential are indeed essential while genes predicted as not essential are not as relevant.

#### Synergistic behaviour of the method

This gene essentiality method finds its success on the synergy between three different factors: 1) biologically relevant gene expression data, 2) a robust prior-knowledge-network (PKN), and 3) the mathematical formulation described in the methods section. Alterations in each of these fundamental pillars affect downstream results increasing the number of false positive predictions. To test the first pillar, biologically relevant gene expression data, we fed the method with “nonsense” expression data by inverting the binary scores obtained from The Gene Expression Barcode 3.0 [22, 23]. This reduced the maximum MCC (starting from a baseline of 0.41 using a ECS of 0.67) to 0.1 (using a ECS of 0.5). To validate the need of a representative prior-knowledge network we repeated the analysis using only the subset of 50 NCI-PID pathways that were labelled as tumorigenic which increased the maximum MCC to 0.53 (using a ECS of 0.67). Finally, we evaluated that this improvement in MCC was only present when the gene expression data was biologically meaningful. To that end, we repeated the analysis using the subset of NCI-PID pathways and the “nonsense” expression data obtaining a MCC of 0.12 (using a ECS of 0.9). The reader should refer to Additional file 2: Table S1 for the complete evaluation. When compared with other state of the art methods [31] our method produces less false positives (Additional file 2: Table S1).

### Case study–breast cancer

Finally, we applied the gene essentiality method to Breast Cancer patient samples [27, 28] and looked for genes significantly predicted as essential in cancer patients using hypergeometric tests. For this purpose, technical duplicates were considered as independent samples. A gene was considered essential for a given patient if ECS > 0. The same procedure was repeated for the different cancer subtypes.

Table 3 shows the top 10 results for the Healthy vs BRCA case while Additional file 3: Table S2 shows the top 10 results for the group-specific comparison. The complete lists can be found in Additional file 4: Table S3 and Additional file 5: S4. In the following lines, we will highlight the relevance of the top 4 (elbow criterion) genes reported in Table 3 with a higher coverage of patients by relying on existing knowledge in the literature.

**Table 3:** BRCA essentiality results

geneID	# pathways	# connections	p.hyper	p.adj	# cancer essential	# total essential	Achilles score
RACGAP1	3	4	3.25E-07	4.91E-05	118	118	− **1.45764**
MIB1	1	3	2.33E-06	1.76e-04	110	110	− 0.34808
EZR	3	4	1.02E-05	5.13e-04	103	103	0.094074
PCNA	2	3	1.94E-05	7.34e-04	126	128	− **1.88689**
TUBG1	1	2	1.40e-04	4.21e-03	88	88	− **1.31599**
CASP3	7	10	5.20e-04	9.81e-03	79	79	0.073582
PKCDELTA	13	18	5.20e-04	9.81e-03	79	79	NA
SDC2	2	25	5.20e-04	9.81e-03	79	79	− 0.06373
BIRC3	4	12	1.68e-03	2.31e-02	89	90	0.149018
GNAI1	1	1	1.68e-03	2.31e-02	70	70	− 0.10703

*geneID* gene identifier in HGNC nomenclature; #pathways: number of NCI-PID pathways where the gene appears; #connections: number of connected genes in those pathways (parents/children); *p.hyper* p-value from a hypergeometric test; *p.adj* adjusted p-value after multiple-testing comparison, #cancer essential: number of cancer samples predicted as essential; #total essential: number of total samples predicted as essential; *Achilles score* average Achilles score across all the BRCA cell lines from the CCLE. Essential genes (Achilles score < − 0.5) are denoted in bold

Number of cancer samples = 153, Total number healthy samples = 11

#### RACGAP1: Rac GTPase-activating protein 1

RACGAP1 is a protein involved in several biological processes including cell cycle, cell division, and differentiation and with a key role in various cellular phenomena including cytokinesis, invasive migration and metastasis. Increased expression of RACGAP1 protein has been previously associated with poor survival as well as significantly associated with increased tumour malignancy in colorectal cancer [32]. It has been shown that its knockdown—in combination with radiotherapy—is associated with a decrease of tumour viability and invasiveness in 4T1 mouse models [33].

#### PCNA: proliferating cell nuclear antigen

PNCA is a protein involved in DNA replication by increasing the processivity of DNA polymerase delta. Immunohistochemical staining of PCNA has been used extensively in breast cancer diagnosis and prognosis [34]. It has been shown that targeting the EGFR/PCNA signalling suppresses tumour growth of triple-negative breast cancer cells [35] and inhibit cancer growth in neuroblastoma and breast cancer mouse xenograft models [36].

#### MIB1: Mindbomb E3 ubiquitin protein ligase 1

MIB1 is a protein that positively regulates Notch signaling by ubiquitinating the Notch receptors, thereby facilitating their endocytosis. It has been shown that MicroRNA-198 suppresses prostate tumorigenesis by targeting MIB1 [37]. MicroRNA-198 also represses cell proliferation and migration and promotes cell adhesion in breast cancer cells [38].

#### EZR: Ezrin

EZR is protein that plays a key role in cell surface structure adhesion, migration and organization. Its inhibition synergizes with lapatinib in a PKC-dependent fashion to inhibit proliferation and promote apoptosis in HER2-positive breast cancer cells [39]. EZR inhibition in hepatocellular carcinoma (HCC) cells decreases their migratory and invasive potential [40].

The reader should note that from the results reported in Table 3, 3/10 genes have a score smaller than − 0.5 (RACGAP1, PCNA, and TUBG1). One of the 10 genes is not included in the Achilles database so 1/3 of the reported genes have scores largely below − 0.5. However, as notice by previous works [1], cell-based gene-essentialities might not share the same core set of essential genes with those identified in vivo in human population studies. The reader should note that the Average Achilles score was included for the sake of providing a more general perspective of each specific gene in the context of cell-lines.

## Discussion

This article introduces a new methodology for the in-silico identification of essential genes which integrates high-throughput gene expression data with predefined biological pathways to provide patient-specific gene essentiality predictions. This method uses a mathematical formulation that identifies the number of lowly expressed genes required to be active for the cell to sustain life, here modelled by the activation of a relevant biological task. This work expands the ideas behind existing CBM-based methodologies going beyond metabolism by considering multisystem networks [18].

We have validated the proposed methodology using a set of 452 cancer cell lines derived from the Cancer Cell Line Encyclopedia where the essential genes had been previously identified using CRISPR knockouts (Achilles Project). When compared to competing methods, our approach identifies essential genes with fewer false positives. Because cell-lines do not represent the entire complexity of cancer, we have further supported the obtained essential genes in an independent breast cancer dataset using existing literature.

The mathematical formulation presented in the methods section makes it possible to have several predictions of essentiality for the same gene. Due to the nature of the problem, initially a single prediction of essentiality was a sufficient condition to consider the gene as essential thus these multiple predictions were summarized into their maximum for each gene. This summarization is very susceptible to false positive results which can have a huge impact in downstream results. We have shown how the integration of multiple predictions into the *Essentiality Congruity Score* (ECS) improves our ability to identify essential genes.

The presented methodology finds its success on the synergy between its three core constituents: biologically relevant gene expression data, a robust prior-knowledge-network that effectively captures cancer biological events and the constraint-based mathematical model described in the methods section.

We have shown that all three elements are necessary by modifying individual constituents. We have proven that missense input data (produced by inverting the discrete expression values) does not yield to valid results. We have also shown that including pathways that do not represent tumorigenic events worsen the essentiality predictions. Finally, we have proven how diluting the impact of false positive predictions derived from the methodology using the ECS further improves the precision when identifying essential genes.

The mathematical formulation described in the methods section distinguishes between highly expressed genes and lowly expressed genes. This discrimination, however, is derived from continuous gene expression data, which was previously discretized using The Gene Expression Barcode 3.0 [22, 23]. This work does not directly tackle this issue, but the selection of discretization strategy can have a tremendous impact on downstream results.

The present methodology assumes that all the actives (abstracts + complexes) included in the PKN are equally relevant for the cell to sustain life. This represents an oversimplification of the reality as not all the actives will affect the cell in the same way. We have shown that removing pathways that do not capture tumorigenic events improve the obtained results demonstrating that there needs to exist harmony between the biological network and the mathematical model. Moreover, we recognise that there are several non-gene related functions that are essential for the cell to survive [41]. In this work, however, we have focused exclusively on essential genes and general essentiality falls outside the scope of this study.

The advent of in-silico approaches predicting essential genes will pave the way for precision medicine by identifying potential drug targets whose deletion can induce death in tumour cells [2]. The work presented here contributes to this direction by providing gene essentiality predictions with single-sample resolution. This has significant competing advantages in a cancer context for example by allowing the identification of genes essential for cancer samples and non-essential for healthy samples or for specific cancer subtypes. However, further efforts are required to develop disruptive in-silico methodologies that accounts for further biophysical knowledge, such as dynamic models or multi-omics data. Overcoming this ambitious challenge will set the foundations for addressing biological questions that were unreachable before.

## Supplementary Information

**Additional file 1:** Supplementary results 1 & 2.

**Additional file 2: Table S1.** manual curation of the NCI-PID pathway list.

**Additional file 3: Table S2.** BRCA group-specific essentiality results (top 10).

**Additional file 4: Table S3.** BRCA essentiality results (BRCA vs healthy) complete list.

**Additional file 5: Table S4.** BRCA group-specific essentiality results (complete list).

## Data Availability

The gene essentiality scores from the Achilles project were downloaded from the DepMap (CRISPR knockouts, CERES method, Version 20Q1): https://depmap.org/portal/download/. The gene expression data for the cell-lines included in the Cell Line Encyclopedia (CCLE) was obtained from the Gene Expression Omnibus (GEO), accession number: GSE36133. The breast cancer dataset used as case study was obtained from the Gene Expression Omnibus under accession number GSE65194. We are current working in an own platform where we will make all our algorithms publicly available and free or charge for academic users. In the meantime, the code to produce the gene essentiality predictions as well as their posterior processing can be made available upon reasonable request.
