# Supplementary material for: ELIMINATOR: essentiality analysis using multisystem networks and integer programming
Source: BMC Bioinformatics. 2022 Aug 6;23:324. doi: 10.1186/s12859-022-04855-z (PMC9357337; doi:10.1186/s12859-022-04855-z)
Supplement: Supplementary file 1 — Additional file 1: Supplementary results 1 & 2. [file 12859_2022_4855_MOESM1_ESM.docx]

# Supplementary Results

**Supplementary Results 1: Toy Example – Solution of the mathematical model**

In the forthcoming lines, we will solve the mathematical model for different scenarios of the toy example included in **Figure 4**. The different scenarios involve different genes highly and lowly expressed. **Table 1** summarises the expression of all the genes and the activity of all the entities included in the pathway for each scenario.

As mentioned in the methods, the methodology comprises two main steps: (i) calculating the minimum number of lowly expressed genes that we need to activate in order to activate a given active ($S_{a}^{wild}$); and (ii) performing an *in-silico* exhaustive gene knockout to find deletions that unavoidably lead to the need of activating extra lowly expressed genes in order to activate the active ($S_{a}^{g}$>$S_{a}^{wild}$). Scenario A will be used to illustrate how the minimum number of lowly expressed expressed genes needed active is calculated. Scenarios B and C will be used to illustrate how essential genes are obtained.

*Scenario A*

In this scenario, FZD7 and FZD1 are highly expressed (𝐺={FZD7,FZD1}) while WNT5A and WNT3A are lowly expressed (𝐿={𝑊𝑁𝑇5𝐴,𝑊𝑁𝑇3𝐴}). The Wnt receptor signalling pathway, planar cell polarity pathway - from now on, the abstract – is the entity required for the cell to sustain life, that is, 𝑨={𝐴𝑏𝑠𝑡𝑟𝑎𝑐𝑡}. Subject to the restriction modelled by equation 7, any valid solution needs for the abstract to be active:

| $E_{a}=1,\forall a\in\boldsymbol{A}$ | 7 |
| --- | --- |

that is, $E_{Abstract}=1$.

The parents of the abstract are two complexes (WNT5A/FZD7, and WNT3A/FZD1) that are connected to the abstract via activation interactions. According to the rules describing the activators presented in the methods section, the activation of the abstract is determined by its activation/inhibition state, which according to equation 5, is defined as:

| $F_{i}=\sum_{\nabla b\in J_{i}} E_{b}-\sum_{\nabla b\in I_{i}} E_{b}$ | 5 |
| --- | --- |

In this case, the abstract (*i*) does not have any inhibitors ($I_{Abstract}= \emptyset$) and has two activators, that is, $J_{Abstract}=\{WNT5A/FZD7, WNT3A/FZD1\}$. Therefore:

| $F_{Abstract}=E_{WNT5A/FZD7}+E_{WNT3A/FZD1}$ | s.1 |
| --- | --- |

According to the equations for activators and inhibitors, the activation of the abstract is defined as:

| $M\cdot\left( E_{i}-1 \right)\leq F_{i}-w$ | 6 |
| --- | --- |

Hence, if we substitute equation s.1 in equation 6 with the standard parameters (M = 1000, w = ½), and bearing in mind that $E_{Abstract}=1$, we obtain:

| $0\leq E_{WNT5A/FZD7}+E_{WNT3A/FZD1}-\frac{1}{2}$ | s.2 |
| --- | --- |

If we clean up the expression above,

| $E_{WNT5A/FZD7}+E_{WNT3A/FZD1}\geq\frac{1}{2}$ | s.3 |
| --- | --- |

$E_{WNT5A/FZD7}$ and $E_{WNT3A/FZD1}$ are binary variables ($E_{WNT5A/FZD7},E_{WNT3A/FZD1}\in\{0,1\})$, thus, for the first expression to be true, at least one of the two complexes needs to be active. The reader should note that the solution where both complexes are active ($E_{WNT5A/FZD7}=E_{WNT3A/FZD1}=1$) is also feasible. In this example, we will solve the equations for the WNT5A/FZD7 complex ($E_{WNT5A/{FZD}7}=1)$ but the reader should know that the same logic can be applied for the WNT3A/FZD1 complex.

The WNT5A/FZD7 complex has two parents that are connected to the child via component interactions. Following the rules for components introduced in the methods section (equations 1 and 2) the activity of a complex is defined as:

| $E_{i}\geq\sum_{\nabla b\in B^{i}} E_{b}-\left( N^{i}-1 \right)$ | 1 |
| --- | --- |
| ${N^{i}\cdot E}_{i}\leq\sum_{\nabla b\in B^{i}} E_{b}$ | 2 |

In this case, $B^{WNT5A/FZD7}=\{WNT5A, FZD7\}$ and $N^{WNT5A/FZD7}=2$. Therefore:

| $1\geq E_{WNT5A}+E_{FZD7}-\left( 1 \right)$ | s.4 |
| --- | --- |
| $2\leq E_{WNT5A}+E_{FZD7}$ | s.5 |

If we clean up the expressions above,

| $E_{WNT5A}+E_{FZD7}\leq2$ | s.6 |
| --- | --- |
| $E_{WNT5A}+E_{FZD7}\geq2$ | s.7 |

Thus,

| $E_{WNT5A}+E_{FZD7}=2$ | s.8 |
| --- | --- |

$E_{WNT5A}$ and $E_{FZD7}$ are binary variables ($E_{WNT5A},E_{FZD7}\in\{0,1\})$, thus this equation is only true when $E_{WNT5A}=E_{FZD7}=1$, that is, both component genes have to be active. As we can see in Figure 4.A, FZD7 is highly expressed but WNT5A is lowly expressed. Therefore, for the abstract to be active, we need at least one lowly expressed gene active (WNT5A). Thus, in this scenario we have three feasible solutions:

- Solution 1: Activate WNT5A ($E_{WNT5A}=1$).
- Solution 2: Activate WNT3A ($E_{WNT3A}=1$).
- Solution 3: Activate WNT5A and WNT3A ($E_{WNT5A}=E_{WNT3A}=1$).

However, not all three solutions are equally good as the objective function of the model defines as the optimal solution the one that minimizes the number of lowly expressed entities active in the final solution (equation 8):

| $S_{a}^{wild}=min\sum_{\nabla i\in\boldsymbol{L}} E_{i},\forall a\in\boldsymbol{A}$ | 8 |
| --- | --- |

Solution 3 requires two lowly expressed genes to be active ($S_{Abstract}^{wild}=2$), thus is a suboptimal solution. Solutions 1 and 2 require one lowly expressed gene to be active, thus they are optimal solutions ($S_{a}^{wild}=1$).

*Scenario B*

In this scenario, FZD7, WNT3A, and FZD1 are highly expressed ($G=\{FZD7, WNT3A, FZD1\}$) while WNT5A is lowly expressed ($L=\{WNT5A\}$). Given that the topology of the pathway is the same, the solution of the mathematical model is analogous to scenario 1 until the calculation of the objective function. In this case, the number of feasible solutions is two:

- Solution 1: Do not activate any lowly expressed genes.
- Solution 2: Activate WNT5A ($E_{WNT5A}=1$).

The first solution is the optimal solution as it does not require any lowly expressed gene to be active, that is, $S_{Abstract}^{wild}=0$).

If we knock-out FZD7 ${(E}_{FZD7}=0)$, the solution that requires WNT5A to be active (Solution 2) is not feasible anymore because following the rules for components introduced in the methods section, the activity of the WNT5A/FZD7 complex is defined as reflected in equation s.8, that is, $E_{WNT5A}+E_{FZD7}=2$.

| $E_{WNT5A}+E_{FZD7}=2$ | s.8 |
| --- | --- |

$E_{WNT5A}$ and $E_{FZD7}$ are binary variables ($E_{WNT5A}, E_{FZD7}\in\{0,1\})$, thus this equation is only true when $E_{WNT5A}=E_{FZD7}=1$, that is, both component genes have to be active. This time, however, we have knocked-out FZD7 ${(E}_{FZD7}=0)$ so this equation will never be true. That is, a knock-out of FZD7 leaves us with one feasible solution:

- Solution 1: Do not activate any non-expressed genes.

The only feasible solution is, of course, the optimal and it does not require any lowly expressed gene to be active, that is, $S_{Abstract}^{g}=0$. Note how $S_{Abstract}^{wild}=S_{Abstract}^{g}=0$. Therefore, in Scenario B, FZD7 is not an essential gene.

*Scenario C*

In this scenario, WNT5A, FZD7, and FZD1 are highly expressed ($G=\{WNT5A, FZD7, FZD1\}$) while WNT3A is lowly expressed ($L=\{WNT3A\}$). Given that the topology of the pathway is the same, the solution of the mathematical model is analogous to scenario A until the calculation of the objective function. In this case, the number of feasible solutions is two:

- Solution 1: Do not activate any lowly expressed genes.
- Solution 2: Activate WNT3A ($E_{WNT3A}=1$).

The first solution is the optimal solution as it does not require any lowly expressed gene to be activated, that is, $S_{Abstract}^{wild}=0$).

If we knock-out FZD7 ${(E}_{FZD7}=0)$, the solution that does not require any lowly expressed genes active (Solution 1) is not feasible anymore. The demonstration has already been shown for Scenario B. This time, the only feasible solution after a knock-out of FZD7 is:

- Solution 2: Activate WNT3A ($E_{WNT3A}=1$).

The only feasible solution is, of course, the optimal and it requires one lowly expressed gene active, that is, $S_{Abstract}^{g}=1$. Note that his time $S_{Abstract}^{wild}=0$ while $S_{Abstract}^{g}=1$. Therefore, in Scenario C, FZD7 is an essential gene.

**Boolean model**

In supplementary section 1, we have seen that for the abstract to be active, at least one of the two gene complexes needs to be active. This intrinsically represents a Boolean equation that can be represented as:

| $E_{Abstract}=E_{WNT5A/{FZD}7}\bigvee E_{WNT3A/{FZD}1}$ | s.9 |
| --- | --- |

Where ⋁ represents the binary operator “or” and *abstract* refers to the Wnt receptor signaling pathway, planar cell polarity pathway.

On the other hand, for either complex to be active, both its component genes need to be active. This can also be represented following Boolean rules as:

| $E_{WNT5A/{FZD}7}=E_{WNT5A}\bigwedge E_{FZD7}$  $E_{WNT3A/{FZD}1}=E_{WNT3A}\bigwedge E_{FZD1}$ | s.10  s.11 |
| --- | --- |

Where ⋀ represents the binary operator “and”.

When substituting equations s.10 and s.11 in equation s.9, the activity of the abstract is directly expressed in terms of the activity of the genes:

| $E_{Abstract}=\left( E_{WNT5A}\bigwedge E_{FZD7} \right)\bigvee\left( E_{WNT3A}\bigwedge E_{FZD1} \right)$ | s.12 |
| --- | --- |

It should be noted that ILP in equation 8 is fundamentally representing a set of Boolean expressions. However, when modelling a standard network, the emerging Boolean rules are too intricate and the use of ILP is required in order to address the problem. This growth in complexity emerges when incrementing the number of nodes and vertices in the network or when feedback loops are included. The reader should note that ILP is not the only option to address the problem as it could also be solved using Logic Programming.

**Supplementary Results 2: Fundamental pillars of the method**

This gene essentiality method finds its success on the synergy between three different factors: biologically relevant gene expression data, a robust prior-knowledge-network (PKN), and the mathematical formulation described in the methods section. The following lines study how alternations in each of these fundamental pillars affect downstream results and how indeed we need all three of them to produce valid results.

*Analysis of gene-expression data*

First, we studied the need of meaningful gene expression data. To that end, we generated a “nonsense” expression dataset by inverting the binary scores obtained from The Gene Expression Barcode 3.0 [1, 2] in the CCLE dataset, that is, substituting the 1s (expressed genes) for 0s (non-expressed genes) and vice-versa (1 → 0, 0 → 1), and repeated the whole analysis for this newly generated dataset. These results are summarized in Figure s.1. shows how for practically any ECS threshold, the dataset with the original expression profile data (original) produces better results than the nonsense dataset (inverse data). This clearly shows how gene expression data drives the obtained gene essentiality predictions and that these are not entirely the result of the network topology.

*Analysis of prior-knowledge-network*

Second, we studied the need of a representative prior-knowledge-network. In the validation dataset, we are comparing Achilles scores between genes predicted as essential and genes predicted as not essential in different of cell lines. These cell lines come from the Cancer Cell Line Encyclopedia, therefore essential means essential for cancer. However, not all the pathways introduced in the NCI-PID database are equally relevant for cancer biology, not all of them represent hazardous effects that can be avoided by knocking-out essential genes.

For example, the “Hypoxia-inducible factor (HIF)-2 alpha” is a hazardous pathway strongly correlated with cancer [3]. On the other hand, the “LKB1 signaling events” is a tumour suppressor pathway [4]. The results for the predictions of essentiality for each of these pathways are summarized in **Table s.1**. **Table s.1** shows how the predictions for the tumorigenic pathway are superior to those of the tumor suppressor pathway. This agrees with the fact that the Achilles scores used in the validation represent essentiality for cancer cell lines. Therefore, it is expected that a tumor suppressor pathway is not going to be able to properly capture this essentiality.

**Table s. 1 - Pathway validation.** Performance of the gene essentiality predictions in two different pathways: Hypoxia-inducible factor (HIF)-2 alpha (HIF-2 A), and LKB1 signaling events (LKB1). The HIF-2 A pathway is a tumorigenic pathway while the LKB1 pathway is a tumor suppressor pathway. N: number of generated predictions after removing globally essential and globally not essential genes, TP: true positives, FP: false positives, FN: false negatives, TN: true negatives

| Pathway | N | TP | FP | FN | TN | MCC |
| --- | --- | --- | --- | --- | --- | --- |
| HIF-2 A | 956 | 476 | 0 | 4 | 476 | 0.9917 |
| LKB1 | 1912 | 2 | 459 | 44 | 1407 | -0.0725 |

In order to evaluate the impact of the PKN on the obtained ECS, we manually annotated (AA & JP, double blinded) the NCI-PID pathways in three different categories: tumorigenic (category 1), tumor suppressor (category 2), and unrelated with cancer (category 3). The complete annotated list is included in Supplementary Table 1. We performed these annotations for the 84 pathways that were included in the analysis. That is, the 92 pathways that did not have any prediction of essentiality for any cell-line included in the validation dataset (static pathways) were excluded from the analysis and thus, the annotation. 50 of these 84 pathways were annotated as tumorigenic (category 1). We hypothesized that including only tumorigenic pathways in the validation dataset would improve the results. Results from this analysis are summarised in Figure s.1. Figure s.1 shows how indeed including only tumorigenic pathways further improves the predictive ability of our method, particularly when the minimum ECS required to define essentiality is high.

**Table s. 2 - Optimal result for each model.** Optimality was defined as the threshold were MCC was maximum. ECS: minimum ECS required to define essentiality. N: number of genes included after removing globally essential and globally not essential genes. Precision: Precision. MCC: Matthew’s Correlation Coefficient. delta.score: average difference in Achilles score between genes predicted as essential and genes predicted as not essential. p-value (t-test): obtained p-value from a t-test comparing the Achilles score of the genes predicted as essential versus the genes predicted as not essential (one-tailed).

| Scenario | ECS | N | Precision | MCC | Delta.score | p-value |
| --- | --- | --- | --- | --- | --- | --- |
| Original | 0.667 | 25 | 0.616 | 0.406 | -0.595 | 6.4032^-246^ |
| Filtered paths | 0.667 | 15 | 0.750 | 0.534 | -0.804 | 0 |
| Inverse data | 0.5 | 30 | 0.227 | 0.095 | -0.092 | 3.53^-35^ |
| Inverse data + filtered paths | 0.091 | 102 | 0.277 | 0.121 | -0.099 | 5.10^-89^ |

For completeness, we also ran this analysis using the nonsense dataset (inverted data + filtered pathways) and saw how selecting only tumorigenic pathways does not improve the results when these come from meaningless biological data (Figure s.1). In each case, we selected the optimal required ECS where the MCC parameter finds its maximum. Table s.2 summarises the results derived from the optimal threshold in each case. We selected the MCC because has been proven
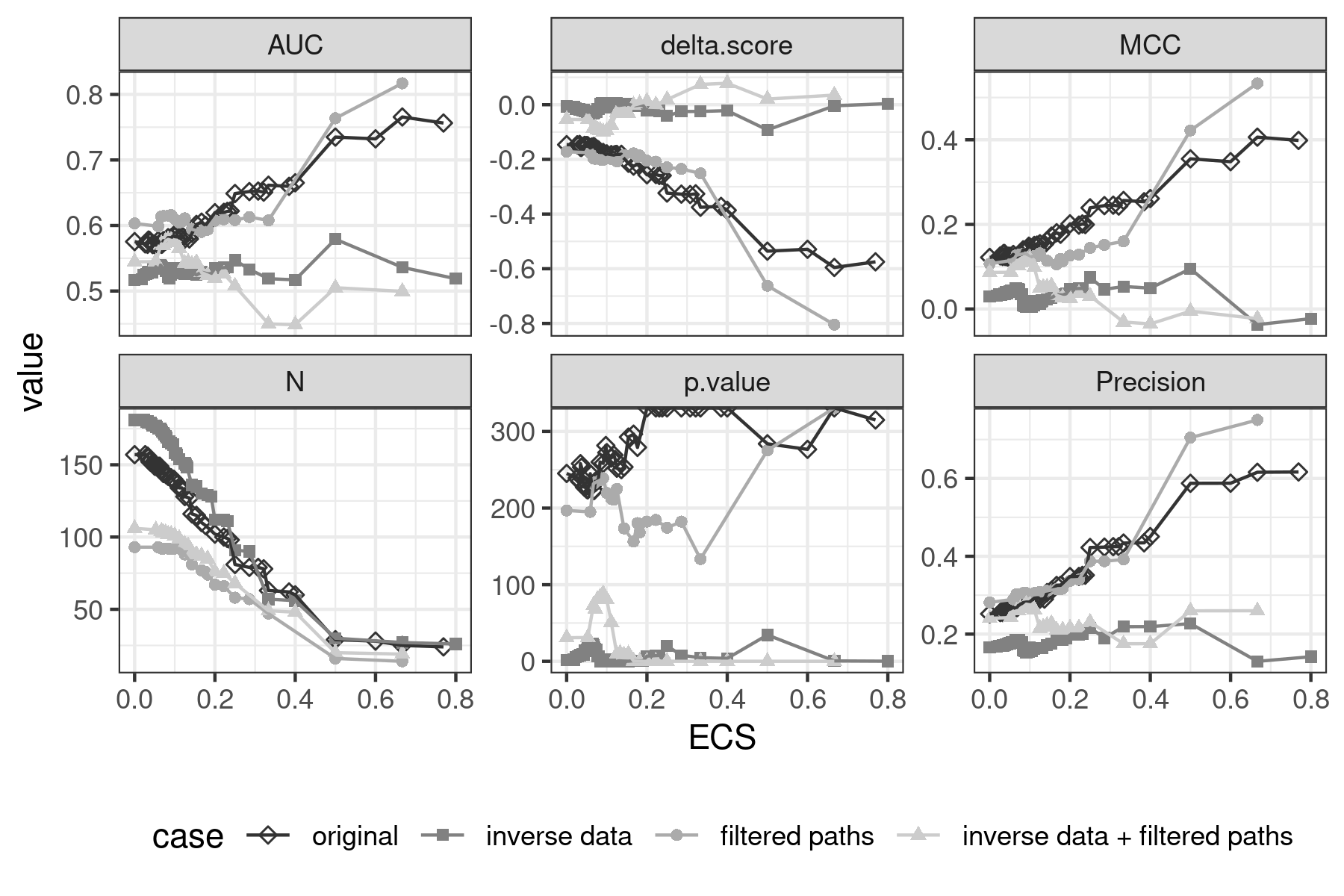
to be the optimal classifier for imbalanced data [5].

**Figure s. 1 - Evaluation of fundamental pillars of the methodology.** Four different models were tested. Original: valid gene expression data and complete set of pathways from NCI-PID; inverse data: nonsense gene expression data (inverted) and complete set of pathways; filtered paths: valid gene expression data and tumorigenic pathways; and inverse data + filtered paths: nonsense gene expression data (inversed) and tumorigenic pathways.

These results further demonstrate the need of all three fundamental pillars in order to produce valid results: biologically meaningful gene expression data, a robust prior-knowledge-network (PKN), and a mathematical formulation valid for the identification of essential genes.

*Comparative with other state of the art methods*

Then, we compared the essentiality predictions obtained using our method with those obtained by another method available in the literature for the identification of essential genes (metabolizer [6]). To this end, we compared the Achilles score of those genes predicted as essential by our methodology (original implementation) with the Achilles score of those genes predicted as essential by metabolizer (t-test). This analysis revealed that the genes predicted as essential by our method have significantly lower Achilles score than those predicted as essential by metabolizer (p.value = 7.7376e-37, delta = -0.2073).

**Supplementary Results 3: Computational time**

CBM approaches are typically computationally intense and require long times to be completed. For an average processor (AMD Ryzen 9 3900X 12-Core Processor), ELIMINATOR takes in average 0.0187土0.0062 seconds per sample/pathway/active/knockout making it compatible with large datasets. For the CCLE/Achilles used in this study, the total CPU time was 20845.41 seconds (5.79 hours). The computational time is proportional to pathway size with large pathways (bigger than 100 nodes) needing 3 times more time to solve (0.0279土0.0054 seconds) than small pathways (smaller than 25 nodes, 0.0096土0.0010 seconds). We want to highlight that this time is per core and the implementation provided is compatible with parallelization.

**References**

[1] McCall MN, Jaffee HA, Irizarry RA. fRMA ST: frozen robust multiarray analysis for Affymetrix Exon and Gene ST arrays. Bioinformatics. 2012 Oct 7;28(23):3153-4.

[2] McCall MN, Jaffee HA, Zelisko SJ, Sinha N, Hooiveld G, Irizarry RA, Zilliox MJ. The Gene Expression Barcode 3.0: improved data processing and mining tools. Nucleic acids research. 2014 Jan 1;42(D1):D938-43.

[3] Leek RD, Talks KL, Pezzella F, Turley H, Campo L, Brown NS, Bicknell R, Taylor M, Gatter KC, Harris AL. Relation of hypoxia-inducible factor-2α (HIF-2α) expression in tumor-infiltrative macrophages to tumor angiogenesis and the oxidative thymidine phosphorylase pathway in human breast cancer. Cancer research. 2002 Mar 1;62(5):1326-9.

[4] Momcilovic M, Shackelford DB. Targeting LKB1 in cancer–exposing and exploiting vulnerabilities. British journal of cancer. 2015 Aug;113(4):574-84.

[5] Boughorbel S, Jarray F, El-Anbari M. Optimal classifier for imbalanced data using Matthews Correlation Coefficient metric. PloS one. 2017 Jun 2;12(6):e0177678.

[6] Cubuk C, Hidalgo MR, Amadoz A, Pujana MA, Mateo F, Herranz C, Carbonell-Caballero J, Dopazo J. Gene expression integration into pathway modules reveals a pan-cancer metabolic landscape. Cancer research. 2018 Nov 1;78(21):6059-72.
